# Supplementary material for: Distinct Cellular Tools of Mild Hyperthermia-Induced Acquired Stress Tolerance in Chinese Hamster Ovary Cells
Source: Biomedicines. 2022 May 19;10(5):1172. doi: 10.3390/biomedicines10051172 (PMC9138356; doi:10.3390/biomedicines10051172)
Supplement: Supplementary file 1 [file biomedicines-10-01172-s001.zip › biomedicines-1701648-supplementary figures.pdf]

Supplementary Material

Distinct Cellular Tools of Mild Hyperthermia-Induced Acquired Stress Tolerance in Chinese Hamster Ovary Cells

Ádám Tiszlavicz [1], Imre Gombos [1], Mária Péter [1], Zoltán Hegedűs [2,3], Ákos Hunya [1], Barbara Dukic [1], István Nagy [2,4], Begüm Peksel [1], Gábor Balogh [1], Ibolya Horváth [1], László Vígh [1] and Zsolt Török [1,*]

[1] Institute of Biochemistry, Biological Research Centre, 6726 Szeged, Hungary; tiszlavicz.adam@brc.hu (Á.T.); gombos.imre@brc.hu (I.G.); peter.maria@brc.hu (M.P.); hunya.akos@brc.hu (Á.H.); dukic.barbara@brc.hu (B.D.); bpeksel@gmail.com (B.P.); balogh.gabor@brc.hu (G.B.); hibi@lipidart.com (I.H.); vigh.laszlo@brc.hu (L.V.)

[2] Core Facilities, Biological Research Centre, 6726 Szeged, Hungary; hegedus.zoltan@brc.hu (Z.H.); nagy.istvan@brc.hu (I.N.)

[3] Department of Biochemistry and Medical Chemistry, Medical School, University of Pécs, 7624 Pécs, Hungary

[4] Seqomics Biotechnology Ltd., 6782 Mórahalom, Hungary

* Correspondence: torok.zsolt@brc.hu

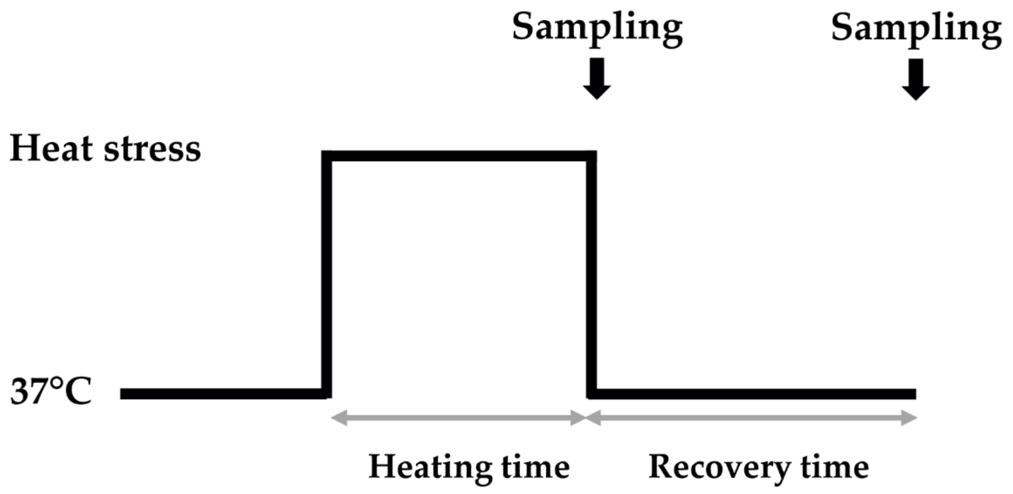

(a)

Recovery time

#	Sample
1	37 °C
2	40 °C, 20 min
3	40 °C, 40 min
4	40 °C, 1 h
5	40 °C, 2 h
6	40 °C, 4 h
7	40 °C, 6 h
8	40 °C, 8 h
9	40 °C, 10 h
10	40 °C, 12 h
11	42.5 °C, 1h (R)

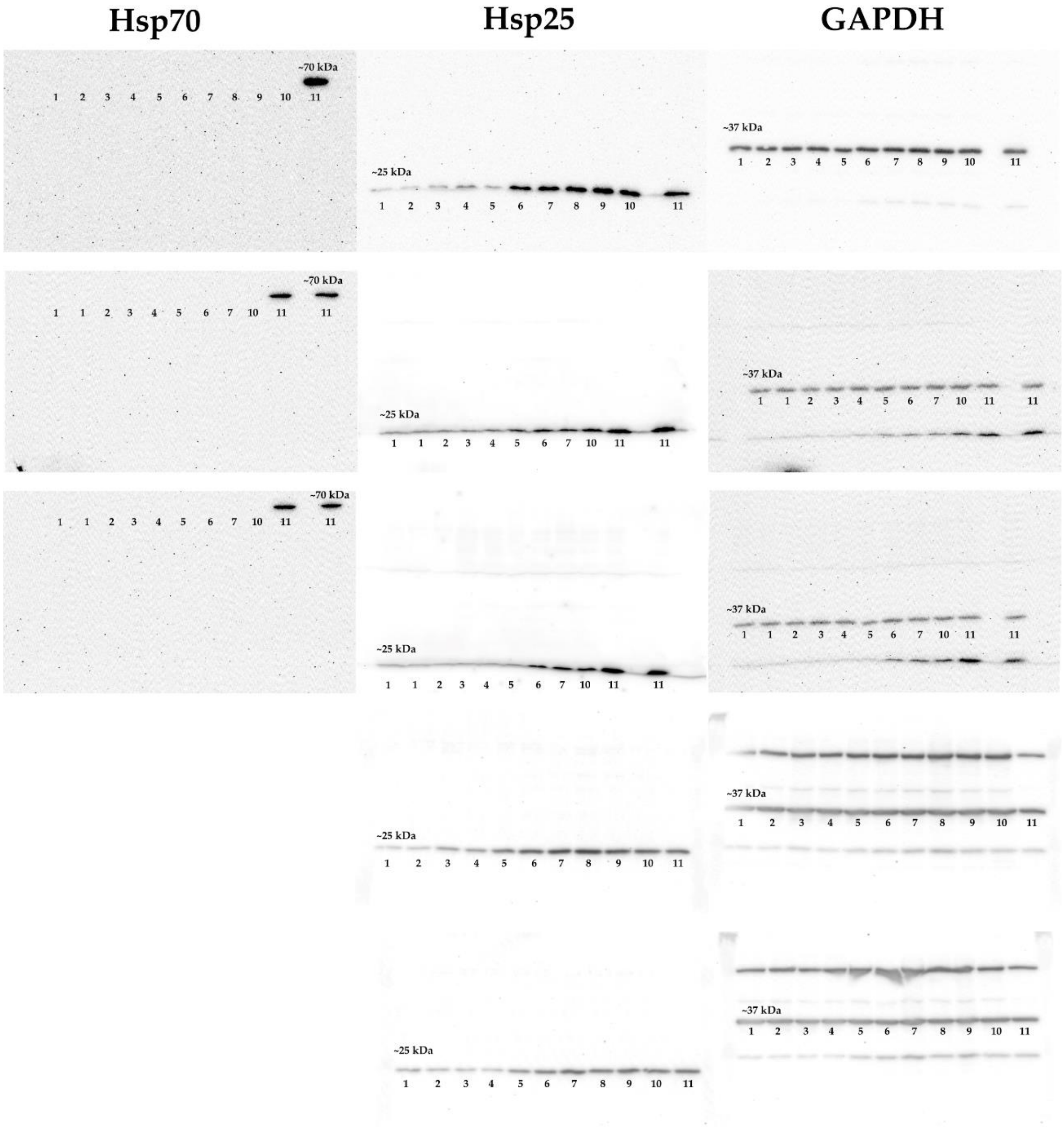

(b)

No Recovery time

#	Sample
1	37 °C
2	40 °C, 20 min
3	40 °C, 40 min
4	40 °C, 1 h
5	40 °C, 2 h
6	40 °C, 4 h
7	40 °C, 6 h
8	40 °C, 8 h
9	40 °C, 10 h
10	40 °C, 12 h
11	42.5 °C, 1h (R)

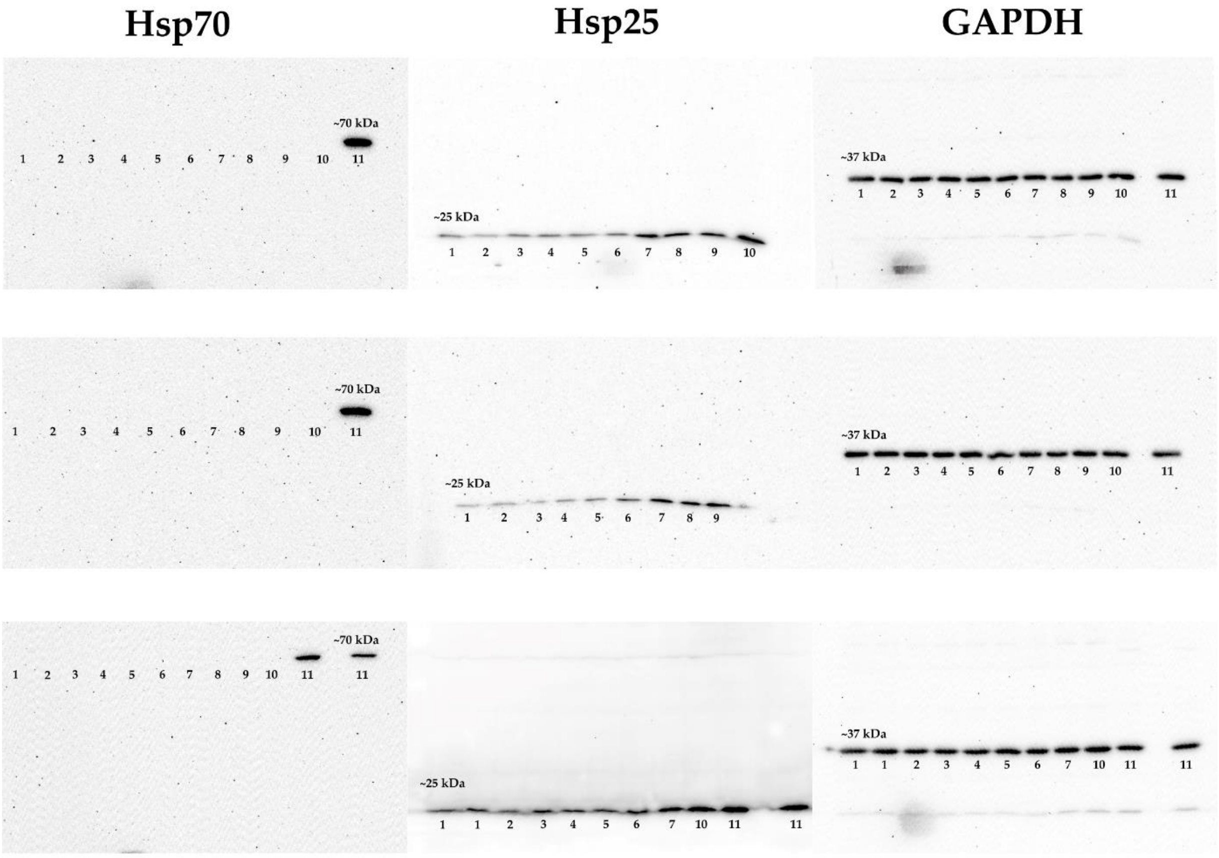

(c)

Figure S1. (**a**) Experimental setup for measuring protein levels (Hsp25, Hsp70, GAPDH) by western blot assay. Supplementary for Figure 1. (**b**) Western blot images of time dependent (0–12h) induction of HSP25 and HSP70 in CHO cells upon 40 °C and 42.5 °C heat treatment. Heat treatment was followed by recovery time (R) prior to sample collection. Each row of blot images represents biological repeats. (**c**) Western blot images of time dependent (0–12h) induction of HSP25 and HSP70 in CHO cells upon 40 °C and 42.5 °C heat treatment. Heat treatment was not followed by recovery time prior to sample collection. Each row of blot images represents biological repeats.

#	Sample
1	37 °C
2	40 °C, 1 h
3	40 °C, 6 h
4	40 °C, 1 h (R)
5	40 °C, 6 h (R)
6	42.5 °C, 1h
7	42.5 °C, 1h (R)

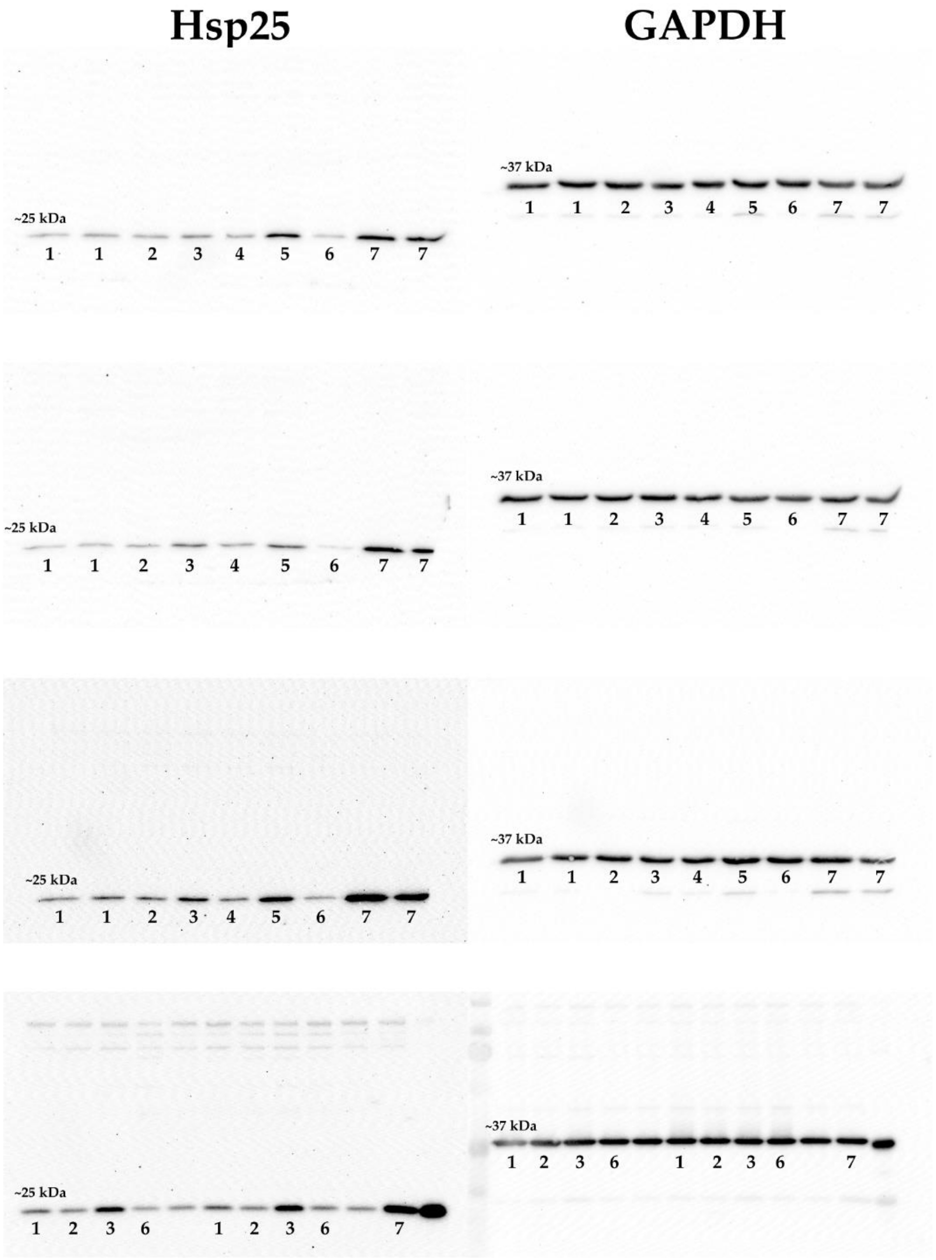

Figure S2. Western blot images of heat shocked (40°C 1, 6 h; 42.5°C 1 h) CHO cells. Treatments were carried out with or without recovery (R). Each row represents biological repeats. Supplementary for Figure 1c,d.

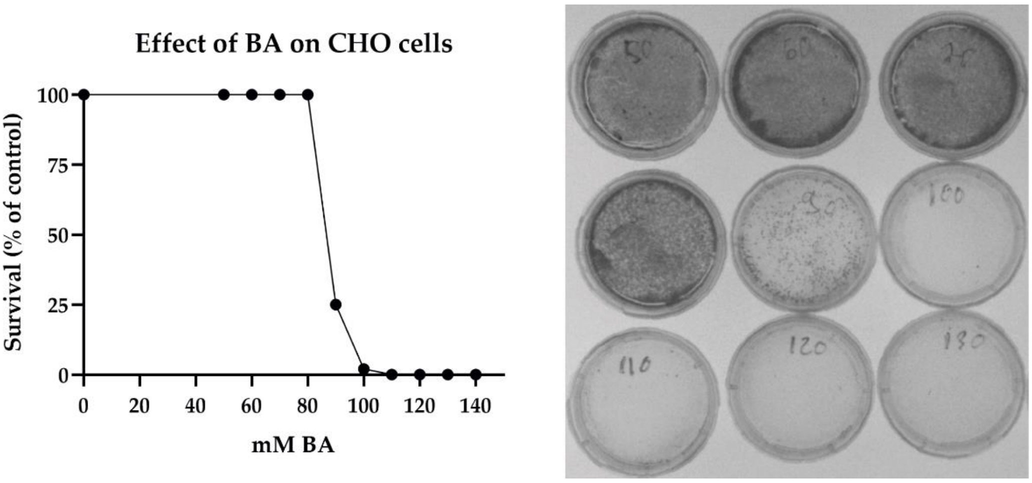

(a)

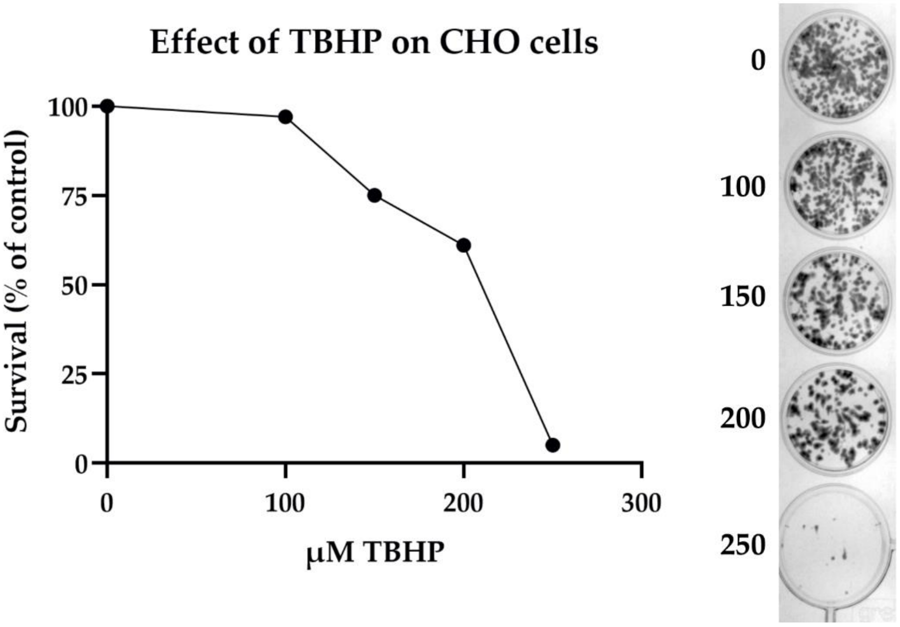

(b)

Figure S3. Cellular survival of non-primed cells treated with different doses of (a) benzyl alcohol (BA) or (b) *tert*-butyl hydroperoxide (TBHP).

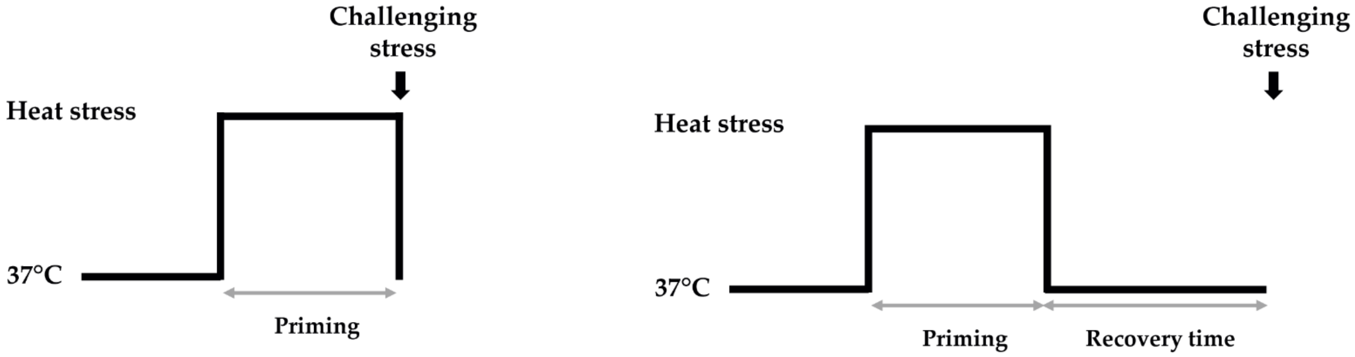

Figure S4. Experimental setup for acquired stress tolerance.

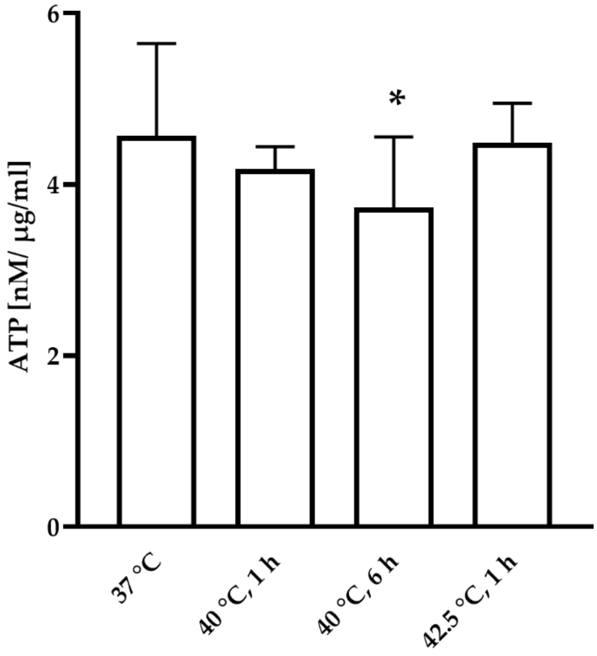

Figure S5. The effect of heat priming on cellular ATP level of CHO cells. ATP level after priming was measured by luciferase-based assay. Cells were primed at 40 °C for 1 h and 6 h and also 42.5 °C for 1 h. All data represent the means ± SD; $n \geq 9$, $p < 0.05$ was considered statistically significant. Paired t-test was used for statistical comparisons in which * denote significant difference compared to 37 °C.

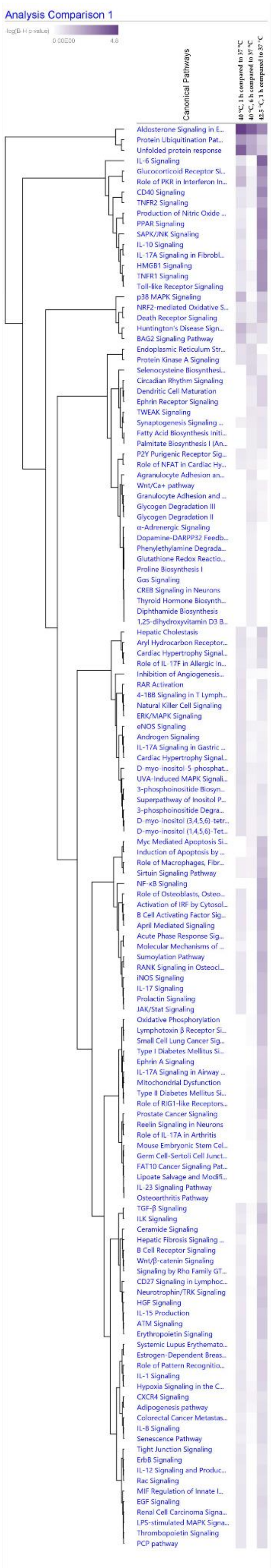

Figure S6. Heatmap representation of canonical pathways significantly affected in heat treated CHO cells. Entries are derived from ingenuity pathway analysis based on significantly altered genes from Table S2.

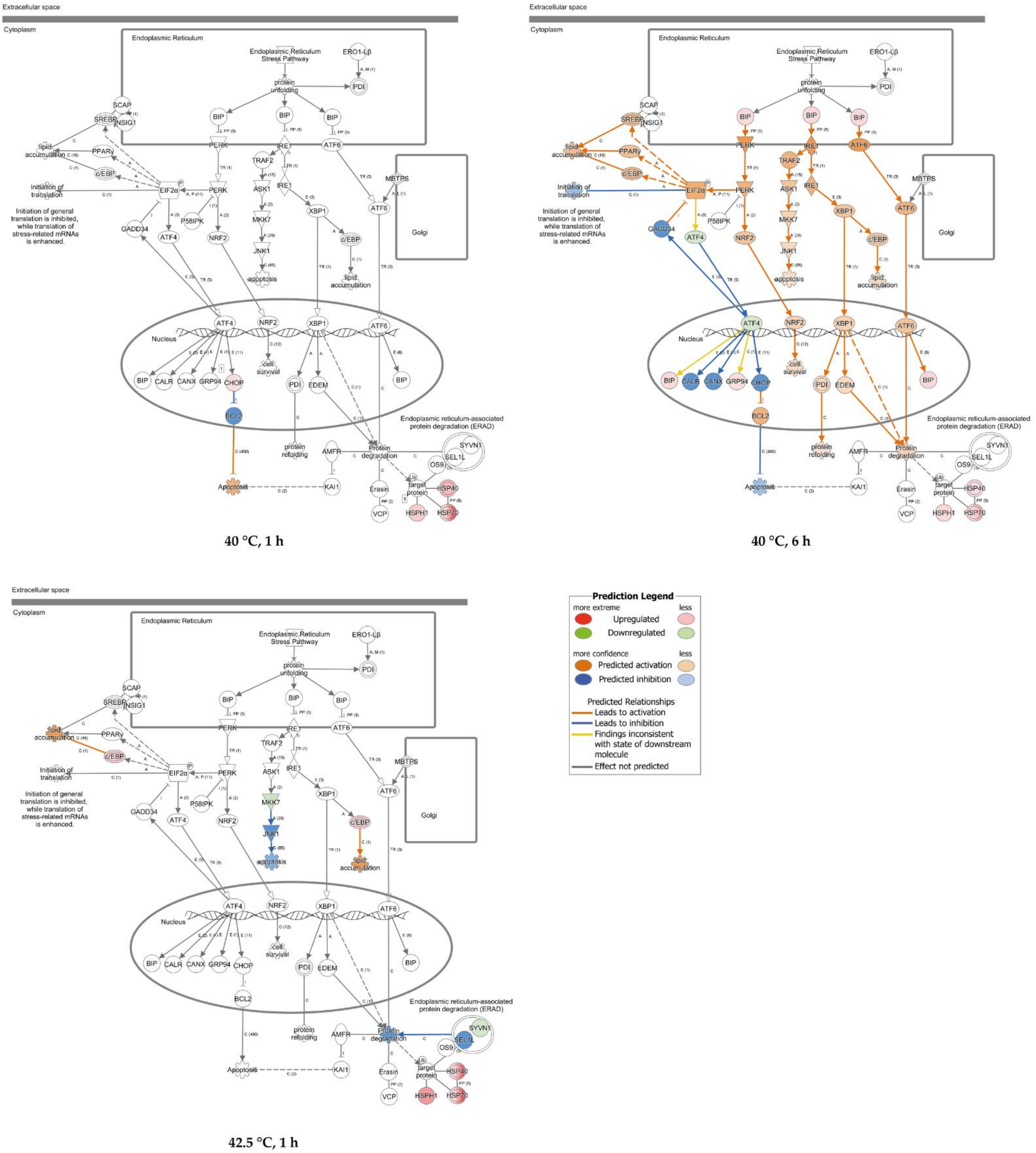

Figure S7. Activation of the Unfolded Protein Response canonical pathway was predicted by the MAP tool of Ingenuity Pathway Analysis (IPA) software based on significant differences in gene expression levels between primed samples and control (40 °C, 1 h; 40 °C, 6 h or 42.5 °C, 1 h compared to control).
